# VegNet: Dataset of vegetable quality images for machine learning applications

**DOI:** 10.1016/j.dib.2022.108657

**Published:** 2022-10-04

**Authors:** Yogesh Suryawanshi, Kailas Patil, Prawit Chumchu

**Affiliations:** a Vishwakarma University, Pune, India; b Kasetsart University, Sriracha, Thailand

**Keywords:** Convolutional neural network, Computer vision, Deep learning, Vegetable classification, Vegetable detection, Vegetable image dataset, Machine learning

## Abstract

The agricultural industry has an unmet requirement for quick and accurate classification or recognition of vegetables according to the quality criteria. This open research problem draws attention to the research scholars every time. The classification and object detection challenges have seen highly encouraging outcomes from machine learning and deep learning techniques. The foundational condition for developing precise and reliable machine learning models for the real-time context is a neat and clean dataset. With this goal in mind, we have developed a picture dataset of four popular vegetables in India that are also highly exported worldwide. In order to generate a dataset, we have taken into account four vegetables: Bell Peppers, Tomatoes, Chili Peppers, and New Mexico Chiles. The dataset is divided into four vegetable folders, including Bell Pepper, Tomato, Chili Pepper, and New Mexico Chile. Further each vegetable folder contains five subfolders namely (1) Unripe, (2) Ripe, (3) Old, and (4) Dried (5) Damaged. The image collection includes a total of 6850 pictures of vegetables in dataset. We firmly feel that the provided dataset is very beneficial for developing, evaluating, and validating a machine learning model for vegetable categorization or reorganization.

## Specifications Table

**Table:** 

Subject	Agriculture Sciences, Horticulture, Vegetable Quality, Machine Learning
Specific subject area	Unripe, Ripe, Old, Dried and Damaged quality image dataset
Type of data	Vegetable images
How data were acquired	The high quality vegetable images were captured using mobile phone camera with different background and artificial light.
Data format	Raw
Description of data collection	The high resolution rear camera of mobile phone was used to capture the different stages of vegetables. The images were taken jpg. Format with the dimension of 4624 × 3472. The captured images then resized to 256 × 256 dimensions using python script. The resized image dataset is stored in four folders viz. Bell Pepper, Tomato, Chili Pepper, and New Mexico Chile. The vegetable images then segregated in five subfolders viz. Unripe, Ripe, Old, Dried and Damaged vegetable according to the vegetables quality. All the images were taken in different light condition with white background. This vegetable image dataset can be used in testing, training and validation of vegetable classification or reorganization model.
Data source location	The dataset presented in this article is prepared at Vishwakarma University, Pune, Maharashtra, India. Latitude and longitude: 18.4603°N, 73.8836°E
Data accessibility	Repository name: VegNet: Vegetable Dataset with quality (Unripe, Ripe, Old, Dried and Damaged) Data identification number(doi): 10.17632/6nxnjbn9w6.1 Direct URL to data: https://data.mendeley.com/datasets/6nxnjbn9w6

**Value of the Data**

• The vegetable dataset contains 6850 high-quality images of four different types of vegetables.
• Vegetable images of Unripe, Ripe, Old, Dried and Damaged levels are included in the dataset.
• This is the first open access dataset of veggies that, to the best of our knowledge, includes Unripe, Ripe, Old, Dried and Damaged quality vegetables.
• This dataset can be used to develop high-quality applications for classifying, counting, and detecting vegetables.
• The dataset can be used by researchers to train, test, and validate their machine learning solutions to classify vegetables as per their quality.
• The dataset can be used to create high-quality vegetable classification apps that are valuable for farmers, the agricultural sector, wholesalers, hawkers, and customers, as well as vegetable export businesses.

## 1 Data Description

As a fraction of all agricultural output, the vegetable market's profit share is sizable [1], [2], [3], [4]. The greatest requirement in the agro-industry is for quick and accurate vegetable classification. Utilizing computer vision and deep learning techniques, the veggies may be divided into many groups based on their outward characteristics, such as shape, size, and color [5], [6], [7], [8], [9]. Vegetables with quality parameters for those that are heavily consumed or exported in accordance with Agricultural & Processed Food Products Export Development Authority (APEDA) are included in this VegNet dataset [10]. This dataset consists of four classes of vegetables namely Bell Pepper, Tomato, Chili Pepper, and New Mexico Chile. This dataset contains the images of these vegetables and not their plants’ leaves. These vegetables are worldwide cultivated by traditional farming, plant tissue culture and hydroponics methods. It is mostly used in culinary and secondary metabolite production [11]. The main reason for choosing these 4 vegetables is the change in color with time. These vegetables contain the red coloured carotenoid 'lycopene' which causes the vegetables to change color when they ripen. This color changing ability will be effective in identifying the stages of vegetables; whether it is ripen, over ripen (old) or dried category.

**Fig. 1:**
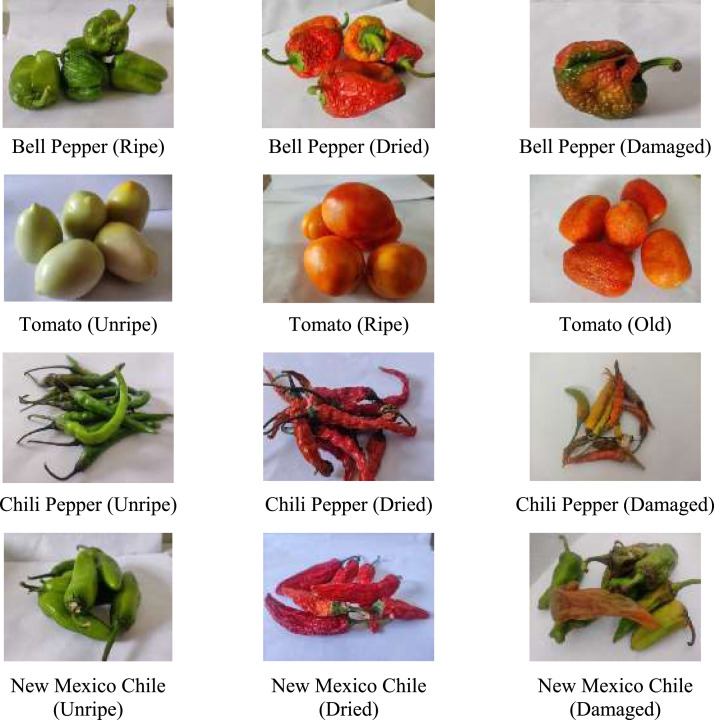
Vegetable images from various quality categories.

In this dataset the images were captured using mobile phone and categorized into five sub-classes namely Unripe, Ripe, Old, Dried and Damaged. Images of vegetables were captured on white backgrounds under various lighting conditions in both indoor and outdoor places. The VegNet dataset contains different folders which are created based on the vegetables quality and not on the image quality. Fig. 1 displays a various photos from dataset's, which were captured in a variety of settings.

## 2 Experimental Design, Materials and Methods

### 2.1 Experimental Design

**Fig. 2:**
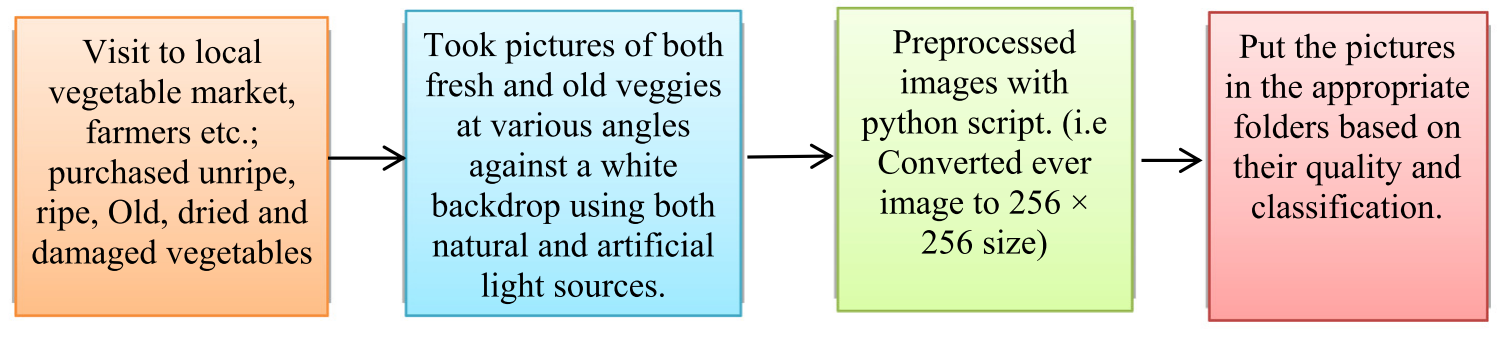
Vegetables data acquisition process.

The high definition rear camera from the Xiaomi Mi-10T was used to capture the photographs of the vegetables. All the 6850 photographs were taken with mobile camera, separated into different categories based on their classification and quality, and then saved in folders. Fig. 2 displays the image data acquisition procedure.

In Table 1, the steps of the data collecting procedure are displayed. From April to June 2022, different angles and backgrounds with natural and artificial lighting are used to photograph the vegetables. A Python script was used to scale all of the dataset photos from their original dimensions of 4624 × 3472–256 × 256. The captured pictures are kept in the.jpg format. The photos taken in a diverse environmental circumstances, including various lighting conditions, a white background, and with various angles. Various researchers used the 256 × 256 dimension for data storage which is helpful to create various machine learning models [12].

**Table 1:** Data gathering process.

Sr. No.	Particulars	Time	Action Details
1.	Data Collection	April - June	Every day, pictures of vegetables in both natural and artificial light, from various angles, and on a white background was taken.

2.	Pre-processing and creating dataset	July	Pre-process the photos using the python script (all images was converted to 256 × 256 resolution). Images then saved in the appropriate folders according to their quality and classification (i.e. Unripe, Ripe, Old, Dried and Damaged)

### 2.2 Materials or Specification of Image Acquisition System

The Xiaomi Mi10T triple rear camera of 64MP+13MP+5MP megapixels resolution were used to take the vegetable pictures. The images were captured with the dimensions of 4624 × 3472. Using a Python script, the original photos, which were 4624 × 3472, were shrunken to 256 × 256 dimension. The captured pictures are kept in the.jpg format. The photos taken in a diverse environmental circumstances, including various lighting conditions, a white background, and with various angles.

Following the image capturing process, the photos were arranged into four files, one for each of the vegetable classes: Bell Pepper, Tomato, Chilli Pepper, and New Mexico Chile. They further divided the categories into five subcategories: Unripe, Ripe, Old, Dried and Damaged. Tables 2 and 3, respectively, present the technical details of the image acquisition devices and the specification of images.

**Table 2:** Specification of image acquisition device.

Sr. No.	Camera Details	Particulars
1	Phone type	Smartphone
2	Smartphone type	Android
3	Company name	Xiaomi
4	Model of Camera	M2007J3SP
5	F-stop	f/1.9
6	Exposure time	1/100 s.
7	ISO Speed	ISO-462
8	Exposure bias	0 step
9	Focal length	5 mm
10	metering mode	center weighted average
11	Mode of flash	No flash, Compulsory
12	Focal length 35mm	25

**Table 3:** Details of acquired images.

Sr. No.	Image details	Image Quality
1	Dimension	256 × 256pixel
2	Width	256 pixels
3	Height	256 pixels
4	Horizontal resolution	72 dpi
5	Vertical resolution	72 dpi
6	Bit depth	24
7	Resolution unit	2
8	Color representation	sRGB

**Table 4:** VegNet Dataset details.

Quality classes	Types of Vegetable classes	Direction of Images while taking the	Type of Backgrounds	Number of each denomination's images	Total No. of Images
Unripe	Tomato, Bell Pepper, Chili Pepper, New Mexico	Front Direction, Top View, Backward Direction, Bottom View, Direction Rotated 180 °	White, Dark color, White light color, Ground, Multicolor	(1) Tomato - 845 (2) Bell Pepper - 52 (3) Chili Pepper - 189 (4) New Mexico Chile- 227	1313

Ripe				(1) Tomato - 955 (2) Bell Pepper - 448 (3) Chili Pepper - 183 (4) New Mexico Chile- 201	1787

Old				(1) Tomato - 1234 (2) Bell Pepper - 349 (3) Chili Pepper - 200 (4) New Mexico Chile- 261	2044

Dried	(1) Tomato - 0 (2) Bell Pepper - 296 (3) Chili Pepper - 593 (4) New Mexico Chile- 500	1389

Damaged	(1) Tomato - 27 (2) Bell Pepper - 31 (3) Chili Pepper - 121 (4) New Mexico Chile-138	317

Total Images in the VegNet Dataset					6850

### 2.3 Method

All the four vegetable Bell Pepper, Tomato, Chili Pepper, and New Mexico Chile were purchased from local market in various stages. The vegetables brought to laboratory and washed it carefully (except dried and damaged). Daily photos were taken using a high definition rear camera of a Xioami Mi10T smartphone with various angles against white backdrops. The images were captured in a single as well as with multiple vegetables. The images were captured with different angle, color, background and lightning situation. Various photographs were captured in the original dimensions, which were 4624 × 3472. Using python script the images then converted to 256 × 256 dimension The created images are publicly available and uploaded online on Mendeley Data [13]. The classes, number of photographs taken, and environments where the images were taken are all listed in Table 4.

## Ethics Statement

The data is available in public. No ethics approval needed for this study.

## CRediT authorship contribution statement

**Yogesh Suryawanshi:** Data curation, Validation. **Kailas Patil:** Conceptualization, Methodology, Software, Supervision, Writing – original draft. **Prawit Chumchu:** Writing – review & editing.

## Declaration of Competing Interest

The authors affirm that they have no known financial or interpersonal conflicts that would have appeared to have an impact on the research presented in this study.

## Data Availability

VegNet: Vegetable Dataset with quality (Unripe, Ripe, Old, Dried and Damaged) (Original data) (Mendeley Data).
